# Spatial analysis of air pollution and childhood asthma in Hamilton, Canada: comparing exposure methods in sensitive subgroups

**DOI:** 10.1186/1476-069X-8-14

**Published:** 2009-04-01

**Authors:** Talar Sahsuvaroglu, Michael Jerrett, Malcolm R Sears, Rob McConnell, Norm Finkelstein, Altaf Arain, Bruce Newbold, Rick Burnett

**Affiliations:** 1 School of Geography and Earth Sciences, 1280 King St West, Hamilton, L8S 4K1, Canada; 2 School of Public Health, University of California, Berkeley, 140 Warren Hall, Berkeley, CA, 94720-7360, USA; 3 Firestone Institute for Respiratory Health, St. Joseph's Healthcare, 50 Charlton Avenue East, Hamilton, ON, L8N 4A6, Canada; 4 Department of Preventive Medicine, School of Medicine, University of Southern California, 1540 Alcazar Street, Los Angeles, CA, 90089-9010, USA; 5 Biostatistics and Epidemiology Division, Health Canada, Tunney's Pasture, Ottawa, ON, K1A 0T6, Canada

## Abstract

**Background:**

Variations in air pollution exposure within a community may be associated with asthma prevalence. However, studies conducted to date have produced inconsistent results, possibly due to errors in measurement of the exposures.

**Methods:**

A standardized asthma survey was administered to children in grades one and eight in Hamilton, Canada, in 1994–95 (N ~1467). Exposure to air pollution was estimated in four ways: (1) distance from roadways; (2) interpolated surfaces for ozone, sulfur dioxide, particulate matter and nitrous oxides from seven to nine governmental monitoring stations; (3) a kriged nitrogen dioxide (NO_2_) surface based on a network of 100 passive NO_2 _monitors; and (4) a land use regression (LUR) model derived from the same monitoring network. Logistic regressions were used to test associations between asthma and air pollution, controlling for variables including neighbourhood income, dwelling value, state of housing, a deprivation index and smoking.

**Results:**

There were no significant associations between any of the exposure estimates and asthma in the whole population, but large effects were detected the subgroup of children without hayfever (predominately in girls). The most robust effects were observed for the association of asthma without hayfever and NO_2_LUR OR = 1.86 (95%CI, 1.59–2.16) in all girls and OR = 2.98 (95%CI, 0.98–9.06) for older girls, over an interquartile range increase and controlling for confounders.

**Conclusion:**

Our findings indicate that traffic-related pollutants, such as NO_2_, are associated with asthma without overt evidence of other atopic disorders among female children living in a medium-sized Canadian city. The effects were sensitive to the method of exposure estimation. More refined exposure models produced the most robust associations.

## Background

Although adverse respiratory health outcomes from exposure to ambient air pollution are biologically plausible, research linking exposure to asthma has been inconclusive [1,2]. Recent research has emphasized the growing contribution and heightened toxic potential of traffic-related air pollution (TAP) near major vehicular corridors [3], as well as significant associations between exposure to TAP and onset of asthma [4]. Other studies have found positive, significant associations between exposure to TAP and adverse respiratory outcomes [5-13], while others have reported null associations [14-16].

The inconsistencies in linking TAP and asthma may be due to exposure measurement error in some studies, which arise partly from the way exposures to traffic pollution are estimated and derived. These exposure estimates include: self-reported traffic density at residence [11,12]; number of cars passing by per 24 hours on the nearest street to a home or school [7,17,18]; distance between the nearest street and home [8,9,16,17,19,20]; identification of the street with highest traffic density relative to a child's school or home [10,21]; perception of residential nuisances related to traffic pollution [22]; indices which combine traffic and distance [14,23,24]; cumulative exposure indices [25,26]; and estimation of pollution exposure at the home using geographic information systems (GIS) and land use regression models [27].

Susceptibility factors have also been suggested to contribute to these observed inconsistencies, including early life exposure [28], duration of residence, parental asthma history, and gender [20,29]. Additionally, Douwes et al[30] suggest there to be a growing importance of investigating specific subtypes of asthma, namely non-atopic or non-allergic asthma. Nystad et al[31] show increasing rates of children with non-atopy related asthma, defined as asthma without atopic diseases (specifically hayfever or eczema), compared to atopy-related asthma. Ronmark et al. [32] report different patterns of risk factors for atopic and non-atopic asthma, defined as asthma with or without at least one positive skin test for type-1 allergy. The separation of atopic and non-atopic asthma is often not considered in the air pollution and asthma literature, and as non-atopic asthma is thought to contribute much of the increased incidence of asthma, it may be accentuating observed inconsistencies in reported results. Further to these complexities, differences exist between the sexes, with girls being more susceptible to asthma than boys [20,33]; despite boys having higher prevalence rates. Age also seems to influence the progression toward onset [34].

In this article, we examine the relationship between within-city or 'intraurban' contrasts in air pollution exposure and childhood asthma in Hamilton, Canada. Further, we test these associations within asthmatic subgroups stratified by the presence or absence of other atopic diseases, gender and age to determine whether these susceptibility factors influence the relationship between air pollution and asthma.

## Methods

### Study area

Hamilton is the ninth largest city in Canada, with a population of over 660,000 in 2001 [35]. The city experiences high levels of pollution exposure for a number of reasons, including traffic and local steel manufacturing plants [36].

**Figure 1:**
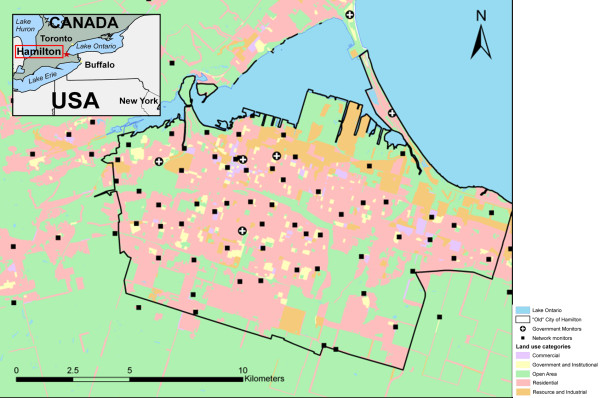
**Locator Map of Hamilton**.

The city has well-documented spatial variability of air pollution [37-40]. Pollution is higher in the major industrial zone located in the northeast and generally lower in the southern and western parts of the city. This is mainly due to prevailing winds, the location of industry upwind of major population areas, temperature inversions that trap pollutants near ground level and topographical elevation created by the presence of the approximately 100 m high Niagara Escarpment [41] (see Figure 1).

Hamilton has been extensively studied in terms of air pollution and mortality, employing ecologic analysis [42], time-series [43,44]; and cohort study designs [39,40]. These techniques have shown that proximity to traffic and higher exposures to ambient air pollution are significantly associated with cardiovascular and stroke mortality rates, but not with respiratory death [41]. Earlier studies investigated the relationship between air pollution and respiratory health in children [45] and in the general population [46], but both studies were inconclusive. Studies of disease prevalence have reported that Hamilton has some of the highest asthma rates for both young adults [47] and children [48] in Canada; however, the association of asthma symptoms with detailed intraurban air pollution exposures has not yet been explicitly tested.

### Study population

The International Study of Asthma and Allergies in Childhood (ISAAC) Phase I questionnaire was administered in 1994–1995 to 6388 children who lived in or around Hamilton [48]. A full description of the ISAAC study protocol has been discussed elsewhere [49]. Briefly, it is a validated tool that uses a standardized questionnaire to assess asthma and respiratory symptoms in children aged 6–7 and 13–14 (pre- and post-pubescent), and allows for uniformity in comparisons across populations [34]. Sampling was based on complete classes from randomly selected schools within the region. Participation rates were 75.1% and 68.6% for the younger and older children, respectively [48]. From this larger sample, 1467 children were selected, based on the geographic extent of the pollution monitoring data available for analysis. Two age groups were tested; those corresponding to grades 1 (ages 6–7) and 8 (ages 13–14) in Canadian schools. Parents filled out the questionnaires for the younger children at home, thereby providing parental consent. The older children completed questionnaires by themselves at school after obtaining written consent from their parents. The questionnaires used were focused mainly on assessing respiratory health status and did not include extensive information on other risk factors. Location of residence was recorded at the level of the 6-digit postal codes, which supply block centroids that are positioned near the front of residential properties.

Children that had answered "yes" to the question "have you ever had asthma" were classified as asthmatic and a variable was created as 'asthma ever'. The same classification ("have you ever had ....") was applied for wheeze and hayfever, creating 'wheeze ever' and 'hayfever ever'. Asthma not associated with hayfever was defined as answering "yes" to asthma ever, but "no" to hayfever ever. Other indicators of probable atopic disease present in the ISAAC questionnaire included the questions "have you ever had eczema" and "have you ever had runny or blocked nose not associated with having a cold or the flu." Similarly, 'asthma without eczema' and 'asthma without runny nose' variables were created for further sensitivity analyses. Variables indicating the presence of 'wheeze ever' and 'current wheeze' (wheeze in the last 12 months) with and without atopy related symptoms were also created. Specific testing for atopy (skin allergen prick tests or serum immunoglobulin E (IgE) levels) was not performed in the Phase I ISAAC study. Hence, to examine the possibility of different effects in atopic and non-atopic asthma, these symptom-related variables were created.

### Air pollution exposure models

We estimated exposure to air pollution using four techniques. First, we created buffers of 50 m and 100 m from major roadways to proxy for traffic pollution exposure based on the DMTI spatial data coverage (DMTI Spatial, Markham, ON). Children living within the specified buffer distance from a major road were assigned the number 1; those who did not were assigned the number 0. Second, we created pollution surfaces for particulates (PM_10_), sulfur dioxide (SO_2_), nitrogen oxides (NO_x_) and ozone (O_3_), using deterministic interpolators applied to three-year averages corresponding to the time of enrolment in the survey. These models were derived from between seven and nine Ontario Ministry of the Environment (MOE) ambient fixed-site pollution monitors located in Hamilton, depending on data availability for the period coinciding with the ISAAC study. Specifically, we derived Theissen polygons, bi-cubic spline and inverse distance weighted (IDW) interpolation techniques [50] for each of the four pollutants.

The third pollution surface estimation method was based on a detailed network of 107 monitoring locations deployed throughout Hamilton for a two-week period in 2002. Passive NO_2 _Ogawa monitors (Ogawa & Co., USA) were set up in duplicate at each location. Every monitor had two filters, yielding four readings per site. Values at each of the 107 locations were based on an average of these four readings. After field retrieval and data cleaning, 100 readings remained available for analysis. Pollutant concentrations from these locations were interpolated to estimate the most likely value of NO_2 _occurring between the monitored locations. We used kriging, an optimal stochastic interpolation method that supplies the best linear unbiased estimate of the variable of interest for this type of exposure calculation [50]. While a temporal difference exists between data collection of the ISAAC study and NO_2 _observations, the spatial trends of pollution in Hamilton between 1995 and 2002 have been relatively consistent, based on annual air quality reports [39]. The stability of the spatial distribution of pollution with Hamilton is also discussed below in terms of the land use regression model.

Our fourth assessment method was a NO_2 _surface created using a land use regression (LUR) model, explained elsewhere in detail [51,52]. Based on the same 100 readings from the passive monitors mentioned above, the LUR model [53] was implemented to assess the land use characteristics, transportation, population and physical geography variables most strongly associated with ambient NO_2 _concentrations. Our final seven-variable model explained 76% of the variation in the measured NO_2_. Variables included: traffic density, open land use within 500 m, industrial land use within 200 m, presence of a highway within 50 m, presence within 1000 m from downtown industrial core, presence downwind from a highway, and distance to the lake. The variables representing traffic density, industrial land use, meteorology, and other activities thought to predict traffic pollution levels had coefficients with the expected sign. Predicted values were used to generate a detailed exposure surface that captured the small-area variability of pollution within the city. Cross validations indicated that the LUR model performed well, demonstrating good predictions for sites not used in model calibration and stable coefficients when assessed with the Chow test [54]. Our seasonal analysis suggested the model was capable of predicting spatial variation within the city for different seasons, probably due to spatial patterns of pollution that remain stable over time [51].

### Confounding variables

Confounding in air pollution and health research occurs when the pollution exposure and the health outcome of interest are correlated and can lead to spurious observed associations. Variations in health outcomes are due to both individual and area, or place-specific, characteristics [55]. Diez-Roux [56] categorized these effects within ecological studies as compositional and contextual confounders. Compositional confounders include those affecting health as a function of the underlying population characteristics (e.g. proportion of smokers). Contextual or area effect variables affect health through the social and economic context of populations. For example, social deprivation in the neighbourhood of residence could exert effects on individual health through a variety of mechanisms [57].

In our analysis, we had limited individual level data on confounders, so we used neighbourhood proxies where available and appropriate. Specifically, we used variables shown to affect the air pollution and health association in previous studies. These included income [39] and dwelling value [37], both of which were obtained from the 1996 Census of Canada with information available at the census tract level. The percentage of smokers [39] has also been shown to affect air pollution and health associations. Our passive smoking data was extracted from a secondary source: a similar asthma study conducted in 1995 among 3369 young adults (aged 20–44 years) at the same time as the ISAAC study, where adults were asked about smoking habits [47]. Sex-specific percentages of 'ever smokers' were calculated and continuous contours were created using the Distance Mapping and Analysis Program (DMAP) point interpolation program [58]. As maternal smoking has been shown to have stronger effects on childhood asthma than paternal smoking [59], children were assigned the smoking value of the female smokers at the contour closest to their residential location.

As our last compositional variables, we used two proxy variables that account for the state of housing and potential exposure to mold: (1) percent of houses built pre-1946 (hereafter referred to as 'percent old houses'); and (2) rate of repair of housing, intended to relate to older home conditions and therefore the increased occurrence of mold [60] and damp conditions [61]. Both of these proxy variables have shown to affect respiratory health. These data were also available from the 1996 Census of Canada.

Our contextual variable was developed from a deprivation index (DI) – a previously created variable shown to be associated with cardiovascular mortality in Hamilton [41]. A similar index was associated with mortality in Quebec, Canada [62]. Social deprivation is a multidimensional construct and relevant variables are often collinear. Because the variables are intercorrelated, and each variable cannot be represented separately, principal components analysis (PCA) is often used to extract the significant dimensions [63]. PCA was run on average income, unemployment rate and low education variables. The DI was created from extracting the first principal component, representing about 80% of the variation in all three variables. The higher the score of the DI, the less favourable was the combination of income, education and employment.

To be included as a true confounding variable rather than an effect modifier, the contextual and compositional variables created had to fulfill three requirements. First, they had to be associated with asthma. Second, variables also had to be associated with pollution exposure. Third, their inclusion in the multiple variable analyses had to change the regression coefficient of the model of asthma related to pollution exposure by more than 10% (see [64] for a similar approach).

### Statistical analyses

To understand the inter-relationships among predictor variables, a correlation matrix was created for the individual pollutants and potential confounders. We conducted bivariate logistic regressions between the dependent variables (health outcomes) and all of the independent variables previously discussed. Significant associations between the pollutants and health outcomes were retained and tested further in trivariate logistic regressions with the contextual and compositional variables. Once the confounding variables were identified, they were entered with the pollution exposures in a series of multiple regression models. Sensitivity analyses conducted for the alternative atopy indicators also used the logistic multiple regression models. We attempted to control for the influence of other confounders with spatial patterns within our data by spatially de-trending to remove the autocorrelation. We did this within a generalized linear model (GLM) structure [65] by applying a natural spline smoother as a sensitivity test for confounding. These analyses were conducted using SPSS version 11.0.1 (SPSS Inc., Chicago, IL) and S-PLUS 2000 (Mathsoft Inc, Seattle, WA).

## Results

The prevalence rates of all asthma and asthma without hayfever are shown in Table 1. With an overall prevalence of 18%, boys had higher prevalence for asthma (21%) than girls (15%), consistent with other Canadian studies on the life course of asthma [66]. The difference between the two sexes was more apparent in the younger age group, with boys having higher rates of asthma without hayfever than girls. Prevalence rates for asthma ever were similar to the larger study from which our population was drawn, but our sub-population had slightly lower rates of wheezing ever for the younger children (data not shown).

**Table 1:** Prevalence of asthma ever, non-atopy related asthma ever without hayfever ever, and hayfever ever

		Asthma		Asthma without hayfever		Hayfever	
	**N**	**n**	**%**	**n**	**%**	**n**	**%**

**All children**	1467	261	18	185	13	220	15

							

**Girls**	729	106	15	76	10	101	14

**Boys**	738	155	21	109	15	119	16

							

**Younger children**	918	115	13	123	13	90	10

**Older children**	549	106	19	62	11	130	24

							

**Younger girls**	465	58	12	47	10	38	8

**Younger boys**	453	97	21	76	17	52	11

							

**Older girls**	264	48	18	29	11	63	24

**Older boys**	285	58	20	33	12	67	24

**Figure 2:**
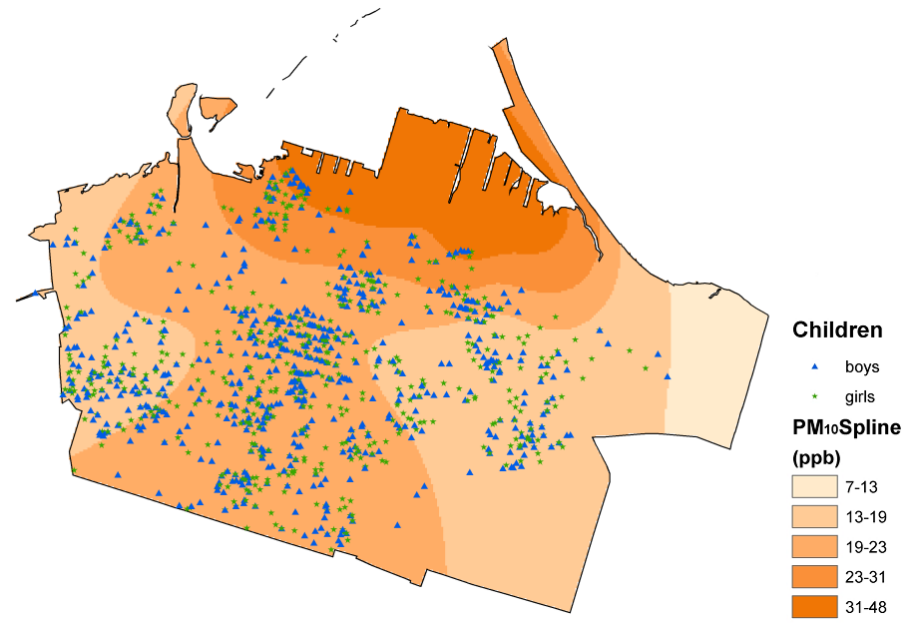
**PM_10 _Spline surface**.

**Figure 3:**
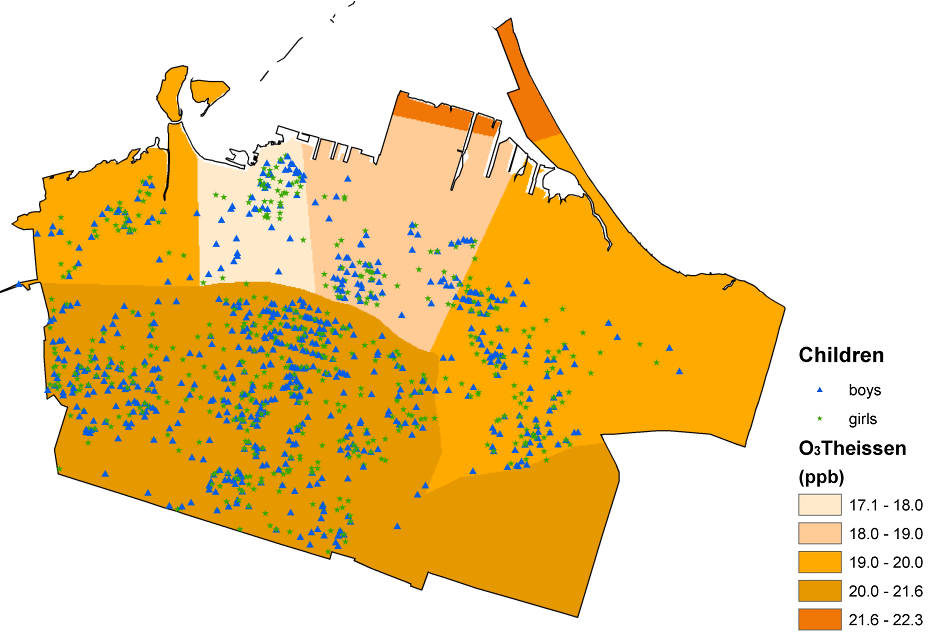
**O_3_Theissen surface**.

**Figure 4:**
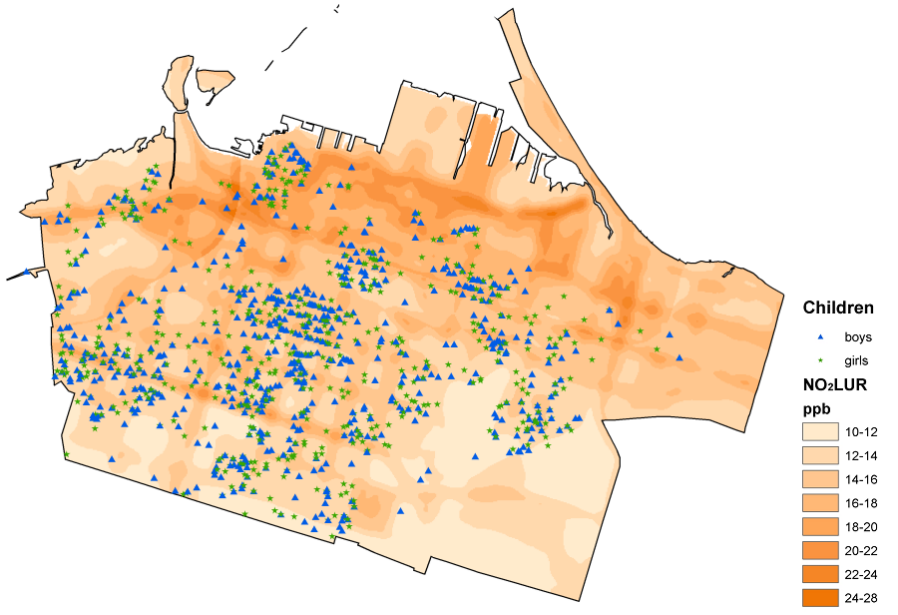
**NO_2_LUR surface**.

In total, sixteen exposure surfaces were created. These included the surfaces derived using the Theissen polygon method for O_3_, NO_x_, and SO_2_, and a splined surface developed for PM_10_. Figures 2, 3 and 4 show the surfaces for PM_10_Spline, O_3_Theissen and NO_2_LUR. All surfaces, except O_3_, showed a pollution gradient within the city that followed the expected trend of higher intensities in the northeast near the industrial core, and decreasing pollution levels towards the outskirts of the city. The O_3 _surface followed the opposite pattern, with lower levels in the downtown area and higher levels at the edges of the city.

The different pollution metrics reflect both different sources and different approaches to modelling exposure. The SO_2_Theissen polygon surface indicates the presence of point-source industries in the northeast end of downtown Hamilton. The Theissen polygon surfaces created discrete categories of pollution levels equal to the measurements obtained at the fixed MOE sites. As these are not smooth continuous surfaces, they may not accurately reflect the real variation of pollution – an inherent unavoidable characteristic of creating such polygons around the monitoring locations [67]. The O_3_Theissen surface had a similar categorizing effect. The PM_10_Spline, on the other hand, may have over-smoothed the true variation of pollution, again, due to the nature of this interpolation technique. Both the kriged and LUR NO_2 _surfaces were based on a denser network of monitors within the city. With the large variation in concentrations measured by the monitors, the kriging methodology was not able to capture the full spatial variation without incorporating some unavoidable errors included in the estimation. The highest errors, however, tended to be outside the area encompassing the children's residence locations. Visually, the NO_2_LUR surface appeared most heterogeneous, with the highest variation occurring around roads and densely populated areas of the lower city.

The concentrations of estimated pollutants were then assigned to the postal code of each child's residential address. Pollution exposures in each group were quite similar for most pollutants (Table 2) with the exception of smaller ranges of PM_10 _exposure for girls and NO_2_LUR exposures for boys. A correlation matrix was constructed for the independent variables (see Additional file 1). The pollutants had low correlations, except for O_3 _with NO_x_, which were inversely related due likely to the scavenging effect of ozone by local sources of NO [68]. As expected, the two measures of NO_2 _were correlated. The DI had a weak positive correlation with the pollutants, except with O_3 _where there was a weak negative correlation. Dwelling value and average income were highly correlated (r = 0.73) and followed very similar patterns in their correlations with pollutants. The rate of repair and percent old houses were also highly correlated (r = 0.72). To avoid introducing multicollinearity, only one of the two variables in each correlated set was retained for the multivariate analysis. DI and smoking did not have strong associations with the other variables, and thus were kept for further testing in the multiple regression models.

**Table 2:** Average and range of pollution exposures

	All subjects		Boys		Girls	
**Pollutants^+^**	**Average**	**Range**	**Average**	**Range**	**Average**	**Range**

PM_10_Spline	20.90	26.98	20.88	26.98	20.92	20.10

NO_x _Theissen	31.77	20.91	31.69	20.91	31.85	20.91

SO_2 _Theissen	5.81	6.04	5.88	6.04	5.74	6.04

O_3_Theissen	20.12	4.30	20.10	4.30	20.15	4.30

NO_2_Kriged	15.36	8.93	15.37	8.93	15.36	8.85

NO_2_LUR	14.84	16.08	14.79	15.55	14.90	12.52

^+ ^Particulate matter in micrograms per cubic meter; gaseous pollutants in parts per billion.

Bivariate logistic regression revealed positive, but insignificant, associations between pollution exposures and asthma outcomes when the whole population was tested. A detailed table is available in the online appendix (see Additional file 2). The odds ratio (OR) for asthma with NO_2_LUR, for example, was 1.02 per ppb (95% CI, 1.00–1.04) but the association was insignificant. Samples were stratified based on literature suggesting that differences exist between the sexes [69], that rates differ by age, and that asthma and asthma without hayfever have different risk factors [31]. When testing the predictive potential of atopy related status for asthma, a positive association was found; namely that children with hayfever symptoms were more likely to have asthma than those without hayfever symptoms (OR = 3.03; 95%CI, 2.20–4.17). After testing interactions between the pollutants, atopy and subgroups, we found effects suggestive of an interaction between hayfever and pollutants in all girls for NO_2_LUR (p = 0.156). The power to test for interactions in epidemiological studies is often poor, resulting in researchers missing important interactions due to lower power [70]. As noted by Selvin [71], relaxing the type 1 error p value from the traditional 5% to 20% is a common approach in epidemiological studies, one that can allow for interaction tests in studies that are not powered for effect modification. In this instance, we had substantive reasons to test for interaction, and the sub-group analysis indicates that girls are more susceptible than boys. Given this, the literature of subgroup interaction and the empirical evidence in our data, we subsequently stratified the sample into subgroups by age, sex, and by age and sex. It is important to note that all subgroups were investigated; however, due to the large number of tested associations between the subgroups, and the voluminous resulting tables, only the significant results for the susceptible groups are included in this paper to avoid detracting from the main findings by listing tables of insignificant effects.

Tables 3, 4, 5 and 6 show the associations for the stratified analysis conducted for the non-atopy related asthma population within the subgroups. Asthma without hayfever was associated with NO_2_LUR for all girls and older girls. We also ran trivariate logistic regressions on the significant associations identified in the bivariate tests for asthma without hayfever (see Table 7). The effects of pollutants remained robust. NO_2_LUR retained significance with asthma without hayfever in all girls for each confounding variable. For the subpopulation of older girls, the odds ratios were generally stronger after adjustment for potential compositional and contextual variables. We used these regressions to test for the effect of the compositional and contextual variables on the percent change in the regression coefficients of the models. Testing the most robust group of associations (NO_2_LUR and all girls) identified the deprivation index (DI) and rate of repair as the variables that reduced the regression coefficient by more than 10%. These were retained as the confounding variables for the multiple variable logistic regressions.

**Table 3:** Odds ratios of bivariate associations between asthma without hayfever and both NO_2_LUR and confounding variables within subgroups of all, younger and older children^+^

	All children		Younger children		Older children	
	Exp(B)	95% CI	Exp(B)	95% CI	Exp(B)	95% CI

**Bivariate Associations**						

NO_2_LUR	1.035	(0.957–1.120)	0.989	(0.889–1.101)	1.100	(0.980–1.234)

Avg Income	0.780	(0.479–1.268)	0.879	(0.470–1.641)	0.591	(0.270–1.296)

Dwelling Value	0.959	(0.905–1.016)	0.971	(0.903–1.043)	0.932	(0.844–1.029)

Rate of repair	1.008	(0.968–1.050)	0.983	(0.930–1.039)	1.012	(0.950–1.077)

Older house	1.002	(0.996–1.009)	0.999	(0.991–1.007)	1.005	(0.996–1.015)

Smoking	1.013	(0.987–1.041)	1.002	(0.970–1.035)	1.009	(0.967–1.052)

DI	1.029	(0.962–1.102)	1.020	(0.925–1.125)	1.047	(0.946–1.160)

**p < 0.05,* p < 0.1

^+ ^calculated for a 1-unit increase in pollutant

**Table 4:** Odds ratios of bivariate associations between asthma without hayfever and both NO_2_LUR and confounding variables within subgroups of all girls and boys^+^

	All girls		All boys	
	Exp(B)	95% CI	Exp(B)	95% CI

**Bivariate Associations**				

NO_2_LUR	1.137**	(1.012–1.278)	0.967	(0.868–1.078)

Avg Income	0.945	(0.465–1.919)	0.658	(0.335–1.294)

Dwelling Value	0.946	(0.864–1.035)	0.969	(0.898–1.045)

Rate of repair	1.043	(0.983–1.108)	1.004	(0.952–1.060)

Older house	1.009	(0.999–1.020)	1.000	(0.992–1.008)

Smoking	1.044	(0.995–1.096)	1.017	(0.982–1.053)

DI	1.049	(0.952–1.156)	1.025	(0.935–1.123)

**p < 0.05,* p < 0.1

^+ ^calculated for a 1-unit increase in pollutant

**Table 5:** Odds ratios of bivariate associations between asthma without hayfever and both NO_2_LUR and confounding variables within subgroups of younger girls and boys^+^

	Younger girls		Younger boys	
	Exp(B)	95% CI	Exp(B)	95% CI

**Bivariate Associations**				

NO_2_LUR	1.072	(0.903–1.272)	0.941	(0.821–1.078)

Avg Income	1.209	(0.475–3.075)	0.724	(0.310–1.688)

Dwelling Value	0.984	(0.875–1.106)	0.970	(0.885–1.062)

Rate of repair	0.960	(0.875–1.053)	0.991	(0.924–1.063)

Older house	1.000	(0.988–1.013)	0.997	(0.987–1.007)

Smoking	0.982	(0.932–1.034)	1.013	(0.972–1.055)

DI	1.063	(0.908–1.245)	0.998	(0.882–1.130)

**p < 0.05,* p < 0.1

^+ ^calculated for a 1-unit increase in pollutant

**Table 6:** Odds ratios of bivariate associations between asthma without hayfever and both NO_2_LUR and confounding variables within subgroups of older girls and boys^+^

	Older girls		Older boys	
	Exp(B)	Exp(B)	Exp(B)	95% CI

**Bivariate Associations**				

NO_2_LUR	1.198**	(1.019–1.408)	1.007	(0.843–1.204)

Avg Income	0.718	(0.244–2.117)	0.472	(0.152–1.464)

Dwelling Value	0.894	(0.772–1.034)	0.966	(0.843–1.108)

Rate of repair	1.062	(0.976–1.156)	1.025	(0.941–1.117)

Older house	1.013	(0.999–1.028)	1.005	(0.990–1.021)

Smoking	1.056	(0.983–1.134)	1.036	(0.969–1.106)

DI	1.032	(0.900–1.183)	1.073	(0.932–1.234)

**p < 0.05,* p < 0.1

^+ ^calculated for a 1-unit increase in pollutant

**Table 7:** Odds ratios of trivariate regressions between asthma without hayfever, NO_2_LUR and confounding variables within subgroups with significant bivariate associations^+^

	All girls		Older girls	
	Exp(B)	95% CI	Exp(B)	95% CI

**Trivariate Regressions**				

NO_2_LUR	1.137**	(1.012–1.278)	1.198**	(1.019–1.408)

+ Avg Income	1.142**	(1.014–1.288)	1.109	(0.935–1.237)

+ Dwelling Value	1.127*	(0.989–1.285)	1.174	(0.964–1.429)

+ Rate of Repair	1.163**	(1.019–1.327)	1.178*	(0.980–1.417)

+ Older house	1.139*	(0.989–1.311)	1.155	(0.948–1.406)

+ Smoking	1.145**	(1.011–1.297)	1.170	(0.967–1.416)

+ DI	1.152**	(1.001–1.326)	1.326**	(1.056–1.666)

**p < 0.05,* p < 0.1

^+ ^calculated for a 1-unit increase in pollutant

We also tested the effect of co-pollutants on our models (see Table 8). For the populations of all girls and older girls, the effect of NO_2_LUR was larger after adjusting for SO_2_, PM_10 _and O_3_. Calculated for a 1-unit increase in NO_2_, the odds ratio for asthma without hayfever among all girls was 1.46 times (after controlling for PM_10_, SO_2_, O_3_, DI and rate of repair), and 2.71 times greater among older girls.

**Table 8:** Co-pollutant models for asthma without hayfever, controlling for DI and rate of repair^+^

	All girls		Older girls	
	Exp(B)	95% CI	Exp(B)	95% CI

				

NO_2_LUR	1.162**	(1.000–1.350)	1.289**	(1.017–1.634)

SO_2_Theissen	1.163	(0.953–1.419)	1.260	(0.832–1.910)

				

NO_2_LUR	1.144*	(0.982–1.331)	1.287**	(1.008–1.643)

PM_10_Spline	1.063	(0.969–1.666)	1.058	(0.918–1.219)

				

NO_2_LUR	1.171**	(1.004–1.366)	1.304**	(1.025–1.658)

O_3_Theissen	1.01	(0.821–1.241)	0.951	(0.685–1.318)

				

NO_2_LUR	1.146*	(0.978–1.334)	1.271*	(0.992–1.627)

PM_10_Spline	1.045	(0.943–1.158)	1.044	(0.891–1.225)

SO_2_Theissen	1.135	(0.912–1.142)	1.246	(0.802–1.934)

O_3_Theissen	1.005	(0.802–1.259)	0.998	(0.691–1.440)

**p < 0.05,* p < 0.1

^+ ^calculated for a 1-unit increase in pollutant

Sensitivity analyses were conducted with a generalized linear model (GLM) with a natural spline smoother [65]. The coefficient changed slightly from 0.128 to 0.130 when the smoother was applied to 10 degrees of freedom (df), and to 0.129 for 20 df (p < 0.05). Using the natural spline smoother, increasing spans indicated a more localized analysis. This sensitivity analysis lends further support to the notion that confounding probably does not bias the coefficients as the effects were robust.

For further sensitivity analysis, we tested the alternative indicators of atopic conditions (eczema and runny nose not associated with a cold) to assess whether the selection of indicator made a difference in the air pollution and asthma relationship. The results were sensitive to the selection of other indicators of atopic conditions, as they were positive for the most part, but were no longer significant.

We also assessed the sensitivity of the association of wheeze ever and current wheeze with the same symptom indicators of atopic conditions, and the pattern of effects was similar to that observed for asthma. After controlling for confounders and copollutants, NO_2_LUR remained significant with wheeze ever without hayfever (OR = 1.13, 95%CI, 1.01–1.23) and current wheeze without hayfever (OR = 1.28, 95%CI, 1.06–1.55) for all girls (OR = 1.15, 95%CI, 1.00–1.31) and older girls (OR = 1.35, 95%CI, 1.10–1.66).

## Discussion

There were no significant associations between any of the exposure estimates and asthma in the whole population, but large effects were detected the subgroup of children without hayfever (predominately in girls). More specifically, after controlling for confounders we observed significant associations between NO_2_LUR and non-atopy related asthma in all girls and older girls. The NO_2_LUR surface provided the only robust associations with all girls and older girls after running the co-pollutant models and GLM sensitivity analyses. Sensitivity analyses for the definition of respiratory symptoms without hayfever showed that using wheeze instead of asthma as a respiratory health effect still revealed similar findings; however, if alternative indicators of atopic disease (eczema or runny nose without a cold) were used, the effects were not robust.

The sizes of the effects found in the asthma without hayfever models are significant. For older girls, the OR is 3.46 (95% CI, 1.19–10.07) for the NO_2_LUR (calculated for mean-minimum pollution). Taking the exposure coefficients from the co-pollutant models brings the estimates closer to OR = 2.98 (95% CI, 0.98–9.02) for older girls and to OR = 1.85 (95% CI, 0.92–3.73) for all girls using the same exposure contrast. The high collinearity between the pollutants probably contributed to the wider confidence intervals in the co-pollutant models. An analysis among a sub-sample with home-based exposure measurements from the Southern California Children's Health Study (CHS) revealed an association between asthma prevalence and home and outdoor NO_2 _of OR = 1.83 (95% CI, 1.04–3.22) per increase of one IQR (5.7 ppb) in exposure [72]. Thus, while both studies observed similarly sized effects, our effects were only within the asthma subgroup without hayfever.

Other researchers have also commented on the relevance and importance of non-atopy related respiratory symptoms. Heinrich and colleagues [73] evaluated TAP exposure using self-administered subjective questionnaires assessing traffic intensity in a population of 6896 adults. High traffic intensity increased the risk for non-allergic asthma, but not for atopic symptoms including allergic sensitization. Non-allergic asthma in this study was identified as having current asthma but a negative screening assay for specific sensitizations to mite, cat, dog, pollen and fungal allergens. Douwes et al. [30] highlighted the importance of investigating non-allergic asthma, as allergic asthma contributes to approximately 50% of asthma cases; thus, much of the increase in asthma incidence may been attributed to non-allergic asthma. Romanet-Manent and colleagues' [74] estimation of the contribution of non-allergic asthma is slightly lower, at 10–30% of asthmatics. Clinical differences between atopic and non-atopic asthma were initially identified by Rackeman et al. [75] to include key factors such as gender, increasing age, increased severity of lung function, and chronic rhinosinusitis. Other recent studies support this earlier differentiation of asthma, placing increasing emphasis on the genetic investigations of each subtype [76].

Douwes et al. [30] also hypothesize that exposures to bacterial endotoxins and air pollutants may induce non-allergic or non-atopic asthma. Nystad et al. [31] suggest that increases in exposure and adjuvant factors may be contributing to the asthmatic response without triggering atopic symptoms, such as hayfever and eczema. Non-allergic asthma is generally associated with increased neutrophil and interleukin-8 levels [77], but the biological mechanisms in which NO_2 _causes the non-atopic effects to occur are not as clear. Particulate pollution has shown to induce neutrophilic air inflammation [78]. Seaton and Dennecamp [79] propose that strong correlations between NO_2 _and ultrafine particles (UFP = particulate matter less than 0.1 micrometre in diameter) may explain why NO_2 _would appear significant in health studies.

In the most stringent analysis controlling for confounders and co-pollutants, effects were observed in all girls and older girls and only for the NO_2_LUR model, a result consistent with recent findings from the CHS cohorts in Southern California [20]. Female sex has shown to increase the risk of a non-allergic type of asthma in an adult population [74] although no mechanism for this difference was suggested. Gold et al. [80] have suggested that gender differences in asthma rates might be due to differences inherent in the mechanical properties of the lung and inflammatory responses. Alternatively, Venn et al. [81] proposed that hormonal changes occurring in early puberty may affect prevalence rates, as well as differential exposures to triggers for wheeze or asthma, such as smoking. Berhane et al. [82] have found that duration and age of onset of asthma differs between the sexes, thus having differential impacts on lung function. There may also be additional factors influencing exposure times to pollution levels that we were not able to account for in this study.

There are limitations in this study that warrant comment. We had few individual-level covariate data for the children; thus, we created proxies for true individual factors by the use of information available at the census tract level. While contextual level data is important [83], lack of control for the individual factors, such as smoking at home, which has shown to impact the respiratory health of children, may have biased our results[16,84]. The sensitivity analysis with the GLM indicated that some confounding might have remained, possibly due to individual level factors with spatial attributes not captured within our proxy variables. The use of the GLM model has, however, reduced this possibility as the removal of spatial structures in the data reduces residual confounding.

Non-atopic asthma is widely-accepted to be identifiable from the absence of positive skin prick tests for IgE mediated sensitizations to common allergens which include dust mites, pet and particular foods [30]. In the absence of this objective evidence relative to atopic indicators, we had to rely on associated symptoms. Upton et al. [85] suggest that clinicians and epidemiologists often use other allergic manifestations such as hayfever to identify atopy when the skin test data is unavailable; indeed they use that same definition to identify 20 year intergenerational trends in prevalence of asthma and hayfever. They do, however, emphasize that the difference between atopic and non-atopic asthma cannot be validated solely on the presence or absence of hayfever. Ronmark et al. [32] defined atopic asthma as asthma with at least one positive skin prick allergy test, while Romanet-Manent et al. [74] defined this as allergic asthma. To avoid adding to these differences and complications, we chose to report our outcome as asthma without hayfever, rather than non-atopic or non-allergic asthma, and acknowledge the fact that we cannot be certain of the atopic/non-atopic difference with the available data.

Since the ISAAC study took place in 1994 and 1995, and the monitoring for the kriging and LUR modelling was conducted in 2002, temporal discontinuity exists between the health outcomes and both of our NO_2 _surfaces. In spite of this potential limitation, research shows that there has been little change in pollution levels within this time period, and, more importantly, that the spatial variation of pollution has not changed substantially from 1995 to 2002 [42]. Hamilton's distinct topography (especially the Niagara Escarpment), downtown industrial core, and prevailing wind direction contribute to a consistent spatial pattern of pollution that has long been known to decrease from the industrial core outwards to the rest of Hamilton [86]. Similar to European findings [87], although pollution levels may fluctuate, the spatial pattern of NO_2 _appears consistent over time.

## Conclusion

We found significant associations between exposure to modeled NO_2 _and asthma without hayfever outcomes in children living in Hamilton. Girls with asthma without hayfever, and particularly older girls, were most susceptible to the effects of NO_2 _or a closely associated co-pollutant. The effects were sensitive to the method of exposure estimation, and more refined exposure models produced the most robust associations. Given the potential role of non-atopic asthma, further studies should include objective determination of atopic status using skin allergy tests or serum IgE levels. Since NO_2 _may proxy for other pollutants that can cause adverse health outcomes such as ultrafine particles [79], further research is needed on the identification and measurement of which pollutants may be associated with the observed effects.

## Abbreviations

CHS: Southern California Children's Health Study; CI: confidence interval; df: degrees of freedom; DI: deprivation index; DMAP: Distance Mapping and Analysis Program; DMTI: Desktop Mapping Technologies Inc; GLM; generalized linear model; IDW: inverse distance weighted; IgE: serum immunoglobulin E; IQR: interquartile; ISAAC: International Study of Asthma and Allergies in Childhood; LUR: land use regression; MOE: Ministry of the Environment; O3: ozone; ON: Ontario; OR: odds ratio; PCA: principal components analysis; ppb: parts per billion; PM10: particulates of 10 micrometers or less; TAP: traffic-related air pollution; UFP: ultrafine particles; USA: United States of America.

## Competing interests

The authors declare that they have no competing interests.

## Authors' contributions

TS participated in study design, conducted the analysis and drafted the paper. MRS provided the ISAAC data, and with RC contributed to expertise and interpretation of data. NF participated in pollution surface creation. AA and BN were both involved in data interpretation and initial study design. RB provided statistical guidance and expertise. MJ participated in study conception and design, data analysis and interpretation, and in writing the paper. All authors have contributed to the overall direction of the research, have made editorial comments, and have read and approved the final manuscript.

## Supplementary Material

Additional File 1 **Supplemental table.** Correlation matrix of independent variables for all study subjects. Click here for file

Additional File 2 **Online appendix.** This online appendix shows the results of all exposure models within different subgroups of the data. Click here for file
